# Co‐Encapsulation of Bioactive Whey Peptides and Probiotics in a Coated Alginate Matrix for the Formulation of a Value‐Added Sports Drink Based on Whey Permeate

**DOI:** 10.1002/fsn3.71255

**Published:** 2025-12-16

**Authors:** Maryam Soltani, Vahid Mofid, Mohammad Rabbani, Seyed Amir Mohammad Mortazavian, Mahdieh Pabast

**Affiliations:** ^1^ Department of Food Science and Industry Islamic Azad University, Science and Research Unit Tehran Iran; ^2^ Department of Food Science and Technology, Faculty of Nutrition Sciences and Food Technology, National Nutrition and Food Technology Research Institute Shahid Beheshti University of Medical Sciences Tehran Iran; ^3^ Department of Chemistry, North Tehran Branch of Islamic Azad University Tehran Iran; ^4^ Nanobiointeractions & Nanodiagnostics, Stituto Italiano di Tecnologia (IIT) Genoa Italy

**Keywords:** antioxidant, encapsulation, protein hydrolysate, sports drink, whey permeate

## Abstract

This study aimed to develop a value‐added, functional hydroelectrolytic beverage based on whey permeate, providing a natural alternative to conventional sports drinks. To achieve this, *Lactobacillus plantarum* , *Lactobacillus casei* , and hydrolyzed whey protein were co‐encapsulated in an alginate (ALG) matrix and coated with whey protein isolate (WPI). The drink samples, containing either ALG or WPI‐coated ALG beads, were evaluated for antioxidant activity, probiotic viability, and sensory attributes following fermentation and during a 28‐day storage period. Additionally, the survival of the encapsulated probiotics under simulated gastrointestinal conditions (SGC) was assessed. The encapsulated whey protein hydrolysate significantly enhanced the antioxidant capacity of the formulated drink samples. Samples containing beads coated with WPI demonstrated a higher antioxidant capacity (15.37% ± 1.5%) after 28 days of storage compared to those with uncoated beads (7.62% ± 1.6%). Probiotics encapsulated in WPI‐coated alginate beads exhibited a significantly lower viability loss during 28‐day refrigerated storage, with a survival rate of 8.61 ± 0.08 log CFU/g, compared to 8.29 ± 0.08 CFU/g in uncoated beads. Furthermore, the WPI‐coated beads enhanced the survival rates of probiotics under SGC, with a survival rate of 7.68 ± 0.08 log CFU/g. The sports drink, formulated with whey permeate and featuring co‐encapsulated whey peptides and probiotics in WPI‐coated alginate, exhibited enhanced probiotic viability and antioxidant potential. The encapsulation process effectively neutralized the bitter taste of the peptides, thereby improving the overall sensory experience of the drink. Given these health benefits, this sports drink could be considered a potential alternative to commercially available sports drinks.

## Graphical abstract

L. plantarum and L. casei were co‐encapsulated with bioactive whey peptides in a WPI‐coated alginate matrix. whey peptides encapsulated in WPI coated ALG enhanced the antioxidant capacity and sensory properties of the sports drink. Probiotic cell survival in SGJ and during storage was enhanced in a whey permeate‐based fermented drink incorporated with coated microcapsules.

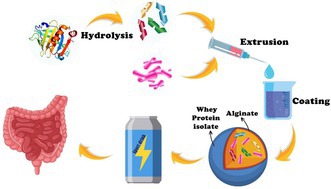

## 1 Introduction

Sports drinks are formulated to enhance hydration by promoting fluid intake, absorption, and retention. Whey permeate‐based isotonic drinks have been proposed as an alternative hydration source to sports drinks due to their higher electrolyte concentrations and comparable carbohydrate content. Whey permeate, a by‐product of the ultrafiltration process of sweet whey, contains lactose as the major constituent, accounting for approximately 90% of the permeate solids, as well as several water‐soluble vitamins that contribute to its nutritional value (Berry et al. 2022).

Fermentation is an effective method for producing functional whey permeate‐based hydroelectrolytes that can reduce lactose content in permeate through lactic acid bacteria (LAB), which is a significant concern for many consumers (Karim and Aider 2022). Moreover, fermented dairy products can serve as an important delivery system for probiotic LAB. These LAB can extend the shelf life of the product by producing inhibitory substances during the fermentation process, which suppress the growth of harmful bacteria (Drgalic et al. 2005).

Recent technological advancements in the formulation of value‐added fermented products, coupled with growing awareness about the use of natural/bioactive ingredients rather than chemicals, indicate that natural ingredients with potent antioxidant activity could be utilized to create innovative functional beverages (Kewuyemi et al. 2022). Incorporating natural/bioactive ingredients into isotonic whey permeate‐based drinks is a viable strategy to obtain functional ingredients and enhance the nutritional value of food products by harnessing the antioxidant health benefits of these ingredients (Servili et al. 2011).

Several studies have underscored the health benefits associated with whey protein peptides, which include antioxidant, immunomodulatory, anticancer properties, and the enhancement of mineral bioavailability (Song et al. 2023). Whey proteins can be modified during processing techniques, such as fermentation and hydrolysis, to enhance their bioactivity and antioxidant properties (Krunic et al. 2018). In the complex process of fermentation, several factors influence the production of bioactive peptides. Usually, the optimal conditions for fermentation and bacterial growth are not ideal for achieving the degree of hydrolysis needed to produce bioactive peptides. To address this, some manufacturers find it more effective to add pre‐obtained bioactive peptides to their products rather than producing them during the manufacturing process. Consequently, in some products, proteins and peptides are added during or after manufacturing to enhance bioactivity and antioxidant properties (Krunic et al. 2018; Mann et al. 2015).

Another important problem that affects probiotic products is the low viability of probiotics during storage and digestion. Encapsulation seems to be an excellent way to protect microorganisms from environmental conditions (Afzaal et al. 2023; Guo et al. 2023). Alginate hydrogels are widely employed in cell encapsulation due to their ease of use, nontoxic nature, and affordability (Burgain et al. 2011). Alginate hydrogels are widely utilized in cell encapsulation due to their ease of use, nontoxic nature, and affordability. However, alginate beads are sensitive to acidic conditions, such as those in the stomach or certain dairy products, and can become unstable when exposed to specific ions or compounds that form chelate complexes. To address these limitations, whey protein coatings have emerged as a promising solution, providing a protective layer that enhances the structural integrity of the alginate microparticles and shields them from external stressors. The electrostatic interaction between the whey protein and alginate promotes the adhesion and stability of the coating, thereby improving the performance of the encapsulation system. Furthermore, the whey protein coating not only enhances the stability and viability of the encapsulated material but also serves as an additional source of protein, which can improve the nutritional value of the final product. The adhesion and stability of the coating are influenced by the electrostatic interaction between the positively charged amino groups of whey protein and the negatively charged carboxyl groups of alginates. This interaction promotes the formation of a stable coating layer, which can protect the encapsulated material from environmental stressors (Pedrali et al. 2023).

Moreover, whey protein coatings can improve the sensory properties of the final product, including taste, texture, and appearance. Due to its neutral taste and odor, whey protein can effectively mask the bitter or unpleasant taste of some bioactive compounds (Krunić and Rakin 2022).

The objective of this study was to formulate a novel whey permeate‐based hydroelectrolyte drink with improved antioxidant capacity, probiotic viability, and sensory characteristics. To achieve this, whey protein peptides and probiotics were co‐encapsulated in alginate‐based carriers and coated with whey protein concentrate (WPC) for added protection. This approach effectively enhanced the functional and sensory properties of the beverage.

## 2 Materials and Methods

Whey protein isolate (WPI) powder containing (> 90% w/w protein) was purchased from ProteinOcean Food Inc. Milk whey permeate, an ultrafiltrated and reverse osmosis concentrated product of cow's skim milk, was obtained from Karaj Ramak Dairy Company (Karaj, Iran). Sodium alginate (medium viscosity) from brown algae was purchased from Sigma‐Aldrich Brazil Ltd. (São Paulo, SP, Brazil). The protease used in this study was pepsin (EC 3.4.23.1, ≥ 3200 units/mg protein; Sigma‐Aldrich Chemie GmbH, USA). Other analytical grade reagents were also purchased from Sigma–Aldrich. MRS Agar culture medium was used for carrying out the microbial test (MERCK, Germany). *L. plantarum* (PTCC 1058) and *L. casei* (PTCC 1608) were obtained from the TAKGENE ZIST Company, Tehran, Iran, with an initial viable cell count of 1 × 10^9^CFU/mL. The MRS agar culture medium was provided by Ibersco (Tehran, Iran).

### 2.1 Hydrolysis of WPI

WPI hydrolysate was prepared by suspending WPI in 10 mM phosphate buffer (pH 7) at a concentration of 15% (w/v). The suspension was stirred and allowed to hydrate and equilibrate to the working temperature of 37°C for 30 min. The suspensions were then adjusted to the enzyme's working pH (pH 2.6 for pepsin) using 2 M HCl or NaOH, respectively. Enzymes were added at an enzyme‐substrate ratio of 1:40 with pH monitoring. The system was stirred throughout the hydrolysis process to prevent sedimentation. The pH was maintained at the working value using 2.0 M HCl (for pepsin) for 3 h. After hydrolysis, the pH was adjusted to neutrality with 2.0 M NaOH or 2.0 M HCl as needed. The hydrolysate was then heated at 90°C for 15 min to inactivate the enzymes. The suspensions were allowed to cool to ambient temperature and then centrifuged at 3500 × g for 30 min. The degree of hydrolysis (DH) was determined by the pH‐stat method (Krunić and Rakin 2022).

### 2.2 Tris‐Tricine Gel Electrophoresis

Tricine‐SDS‐PAGE electrophoresis was used to separate peptides based on their molecular weight. In this experiment, the hydrolysate was subjected to Tris‐Tricine gel electrophoresis. The technique utilized an acrylamide/bisacrylamide mix (49.5% T; 3% C) in a Mini Dual Vertical Electrophoresis Unit with a maximum voltage of 500 V and a maximum temperature of 45°C. Anode buffer (0.2 M Tris, pH 8.9) and cathode buffer (0.1 M Tris, 0.1 M Tricine, 0.1% SDS, pH 8.2 ± 0.2) were used during the procedure to facilitate the separation of peptides based on their molecular weight (Kheroufi et al. 2022).

### 2.3 The Antioxidant Capacity of WPI and WPH

To assess the antioxidant capacity, WPI, and whey protein hydrolysate (WPH) were tested using the DPPH radical‐scavenging activity assay. Briefly, test samples (10 mg/mL) were combined with 0.1 mM methanolic DPPH solution in a 1:1 ratio. The mixture was then vigorously shaken and incubated for 30 min at room temperature in the dark. Following incubation, the absorbance of the resulting solution was measured at 517 nm. The scavenging effect was quantified using the following equation:

$$\begin{array}{c} \%\text{DPPH radical scavenging activity}\\ \;=\left({\mathrm{Abs}}_{\text{control}}-{\mathrm{Abs}}_{\text{sample}}\right)/{\mathrm{Abs}}_{\text{control}}\times 100, \end{array}$$

where Abs control and Abs sample are the absorbance values of the blank sample and DPPH assay solution, respectively, at 517 nm. The measurements were performed in triplicate (Gao et al. 2010).

### 2.4 Inoculum

The probiotic cultures of *L. plantarum* and L. casei were inoculated in MRS broth and incubated at 37°C for 48 h. The cultures were then centrifuged for 15 min and washed with 0.85% NaCl solution. The cells were subsequently centrifuged at 2370 × g for 10 min at 4°C, washed twice with 10 mM phosphate buffer (pH 7.0), and resuspended in saline solution to obtain a solution containing approximately (1.5 × 10^9^9^CFU/mL).

### 2.5 Microencapsulation and Coating

ALG microparticles were obtained using an electrostatic extrusion technique with adaptations. Sodium alginate solutions were prepared in distilled water (1.5% w/w) and pasteurized in a water bath at 60°C for 60 min. The solutions were then cooled overnight at room temperature. A mixture of probiotic culture and WPH, in a weight ratio of 1:1, was dispersed in a sodium alginate solution, in a weight ratio of 1:4, at a temperature of 30°C. The encapsulated bacterial cells were prepared using an Inotech IE‐50R Encapsulator (Inotech AG, Dottikon, Switzerland) equipped with an 80‐μm nozzle and a syringe pump loaded with 15 mL of feed solution containing the microbial cells, following the method provided by Hébrard et al. (2010) with certain modifications. The instrument was operated at a 5 mL/min feed rate. The microparticles were solidified in 200 mL of 5% (w/w) calcium lactate under stirring for 30 min. Finally, the suspensions containing the beads were collected in sterile flasks, washed and filtered with ultrapure water, resuspended in 100 mL of phosphate buffer (10 mM, pH 7.0), and stored at 4°C (Hébrard et al. 2010).

Beads were coated with WPI by immersion. WPI was rehydrated in deionized water (10% w/w) by gentle magnetic stirring for 1 h at room temperature and, after adjusting the pH to 7.0, pasteurized at 78°C for 45 min to completely denature the proteins. ALG beads harvested from the calcium lactate solution were stirred for 10 min in denatured WPI solution (10% w/w) and then transferred to a 6% (w/w) calcium lactate solution for 5 min. Beads were collected and rinsed with deionized water.

### 2.6 Encapsulation Efficiency (EE)

The encapsulation efficiency was calculated according to the following equation as proposed by Pitigraisorn et al. (2017):

$$\mathrm{EE}\%=\left(N/N_{0}\right)\times 100$$

where N is the number of viable cells (log CFU/g) released from the beads and N_0_ is the number of free viable cells (log CFU/g) in the feed solution before the encapsulation process.

### 2.7 Characterization of ALG and WPI‐Coated ALG Beads

The morphology of the beads was observed using an optical microscope. The average diameter of the beads was determined using a Mastersizer 2000 (Malvern Instruments, Southborough, MA, USA). The measurements were performed at 25°C.

### 2.8 Production of Fermented Sports Drink

Concentrated ultrafiltrated permeates were placed in 250 mL Erlenmeyer flasks containing samples that were inoculated by adding 1% (v/v) of either (i) WPH/probiotics encapsulated in alginate beads or (ii) WPH/probiotics encapsulated in WPI‐coated alginate beads. Samples were incubated at 42°C. An incubation time of 4–4.5 h was considered for lowering the pH to 5.00. Subsequently, the products were refrigerated to below 5°C and kept under these conditions for 28 days.

### 2.9 The Antioxidant Capacity of Sports Drink Samples

The antioxidant capacity of the carrier and whey‐based substrate was examined for drink samples with uncoated and WPI‐coated ALG beads during fermentation and storage. To evaluate the drink samples' antioxidant capacity, the whey permeate‐based substrate was filtered through a membrane with 0.7 mm pores, and the resulting permeate solution was collected and assessed. The remaining beads on the membrane were washed with a physiological solution and analyzed for their antioxidant capacity. For the beads, their antioxidant capacity was determined by dissolving 1 g of beads in 4 mL of a 2% sodium citrate solution. The resulting solution was then subjected to analysis using the DPPH method to determine its antioxidant activity.

Each sample, consisting of either 1 mL of substrate or 1 mL of dissolved beads, was mixed with methanol in a 1:4 ratio. Subsequently, the mixture was subjected to centrifugation at a speed of 6000 rpm for 10 min. Following centrifugation, 1 mL of the resulting supernatant was combined with 1 mL of methanol and 1 mL of a 0.1 mM DPPH free radical solution. The absorbance of the sample was determined using a UV spectrophotometer (Unico, S 2100 SUV, Dayton, NJ) at 517 nm. All analyses were performed in triplicate. The DPPH radical scavenging activity percentage was calculated as follows:

$$\begin{array}{c} \%\text{DPPH radical scavenging activity}\\ \;=\left({\mathrm{Abs}}_{\text{control}}-{\mathrm{Abs}}_{\text{sample}}\right)/{\mathrm{Abs}}_{\text{control}}\times 100, \end{array}$$

where Abs control and Abs sample are the absorbance values of the blank sample and DPPH assay solution, respectively, at 517 nm. The measurements were performed in triplicate (Gad et al. 2013).

### 2.10 Stability of Fermented Sports Drink Samples

Fermented drink samples were collected every 7 days and analyzed for viable cell count, pH value, and titratable acidity over a 28‐day storage period. The pH value was measured at room temperature using a Metrohm pH meter (Switzerland). Titratable acidity was determined using the Soxhlet–Henkel method (Legarová and Kouřimská 2010). Cell numbers of *L. casei* and *L. plantarum* were determined by the pour‐plate counting method on MRS agar. The cell numbers were quantified and expressed as log_10_ CFU/mL for free cells and log_10_ CFU/g for encapsulated cells. To release the encapsulated cells, 1 g of beads was added to 9 mL of a 2.0% (w/v) sodium citrate solution (Varga 2006).

### 2.11 Survival of Free and Encapsulated Probiotics in Simulated Gastrointestinal Conditions

Bacterial viability in a gastric simulation medium was examined at pH 1.5 ± 0.02. To simulate the gastric environment, pepsin was mixed with a 0.2% sodium chloride solution, and the pH was reduced to 1.5 using 0.08 M hydrochloric acid. The solution was filtered through a 0.2 mm membrane and sterilized for 15 min at 121°C. Each sample (0.5 g) was incubated in a sterile flask containing 4.5 mL of acidic solution at 37°C for 2 h, followed by centrifugation at 11.95 g for 10 min. After removing 0.5 mL of the supernatant and preparing serial dilutions, the samples were cultured on MRS agar medium with 0.1% peptone water diluent and incubated at 37°C to determine the survival of free and encapsulated probiotic bacteria (Gamage et al. 2016).

### 2.12 Sensory Evaluation

The sensory characteristics of the formulated sports drink samples were evaluated using a sensory acceptability test. Different samples were placed in 10 mL glass containers and coded randomly to prevent bias. Sensory evaluation of samples after 1 night of storage at 4°C was performed by 12 trained panelists using a hedonic scale (5 points for “like extremely” and 1 point for “dislike extremely”). A descriptive test for taste, odor, thirst‐quenching ability, and overall acceptability was also performed by the panelists (Stokes et al. 2017).

### 2.13 Statistical Analysis

To determine if there were significant differences between the means of the obtained data, ANOVA and Duncan's multiple range test were used with SPSS software. A significance level of *p* < 0.05 was used for all analyses, and differences at this level were considered statistically significant.

## 3 Results and Discussion

### 3.1 Hydrolysis Degree, SDS‐PAGE Analysis, and Antioxidant Capacity of WPI and WPH

The degree of protein modification by pepsin was estimated by analyzing the degree of hydrolysis (DH), with the findings presented in Table 1. WPH displayed a DH of 12.15% ± 0.41%.

**TABLE 1:** Degree of hydrolysis of WPH and DPPH scavenging capacity of WPI and WPH.

	Whey protein isolate (WPI)	Whey protein hydrolysate (WPH)
DH (%)	—	12.15 ± 0.41^a^
DPPH (%)	12.11 ± 1.05	23 ± 1.25^b^

*Note:* Means followed by different lowercase letters differ statistically in column (*p* < 0.05). The values obtained are the means ± standard deviation of triplicates.

**FIGURE 1:**
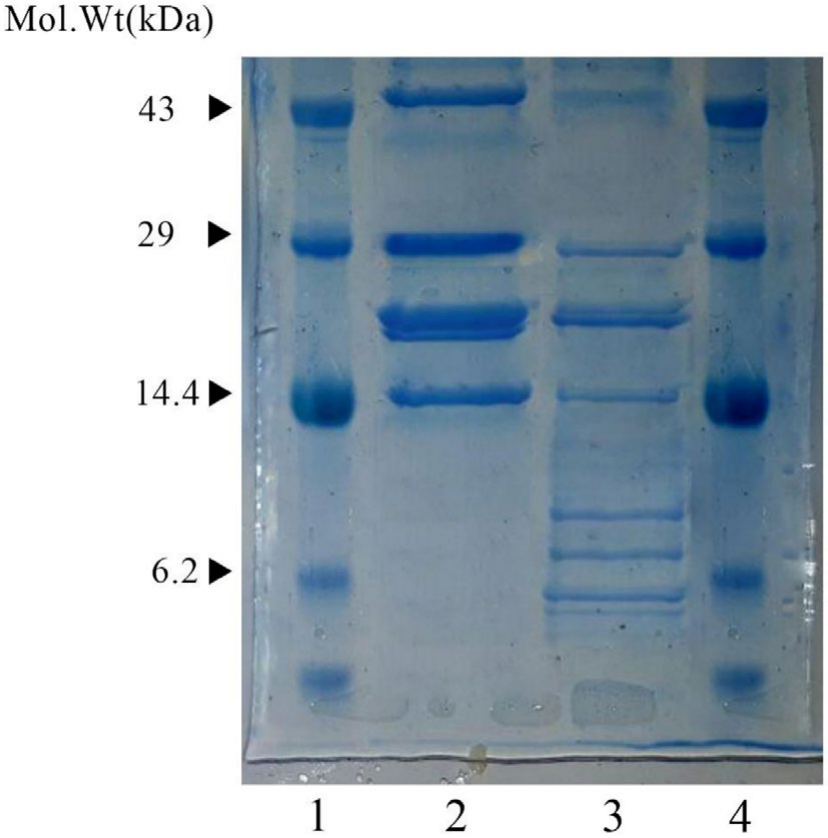
Tricine SDS‐PAGE of whey protein hydrolysates from a 15% protein solution. Lane 1—Standard; lane 2—whey protein isolate; lane 3—whey protein hydrolysate of DH 12.15%; lane 4—Standard.

To investigate the alterations in the fractions of WPI after hydrolysis and the subsequent formation of low‐molecular‐weight peptides, Tricine SDS‐PAGE analysis was conducted on both WPI and WPH. Figure 1 illustrates the results of the Tricine SDS‐PAGE analysis. The peptide profiles obtained from the hydrolysis of 15% WPI (w/v) solutions revealed the presence of low‐molecular‐weight peptides measuring < 10 kDa (Figure 1, Lane 3). Unhydrolyzed WPI did not display any of these low‐molecular‐weight peptides (Figure 1, Lane 2). Bioactive peptides have a size range that includes peptides with molecular weights less than or greater than 10 kDa.

The antioxidant activity of WPI and WPH was evaluated using the DPPH radical scavenging activity assay. The results indicated an antioxidant activity of 23% ± 1.25% for WPI and 12.11% ± 1.05% for the hydrolysate. It was observed that the antioxidant capacity of whey protein significantly increased after hydrolysis (*p* < 0.05). Previous studies have also reported the potential antioxidant activity of WPH, with pepsin‐hydrolyzed protein displaying the highest activity (Balawejder et al. 2020; Olbrycht et al. 2020; Osman et al. 2021).

### 3.2 Characterization of Encapsulation Efficiency (EE%), size, and Morphology of Beads

**FIGURE 2:**
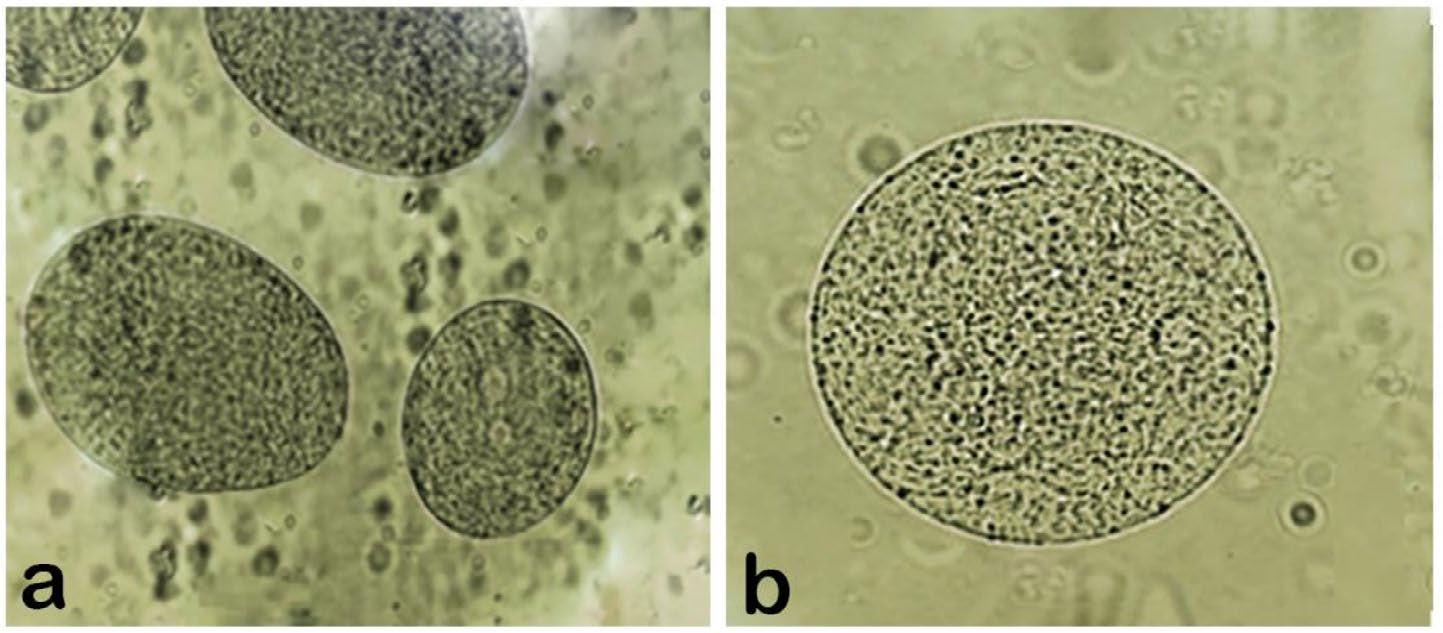
Optical microscopy of (a) alginate beads and (b) WPI‐coated alginate beads.

The morphology of the beads is shown in Figure 2. The size of both the coated and uncoated beads, as determined by Mastersizer‐2000, was approximately twice the diameter of the feed nozzle used in these experiments. The beads appeared spherical with a compact surface. Coating the ALG beads with WPI increased their diameter by approximately 70 μm (Table 2) and provided a uniform covering on the surface. It is essential for the bead to have a diameter of 40–100 μm to ensure the survival of the microencapsulated probiotic without affecting the sensory properties of the food product (Xia et al. 2020). However, to protect probiotics during transit through the gastrointestinal tract at the low pH of gastric juice, only microparticles with a diameter above 100 μm have been proven effective (Hébrard et al. 2010).

**TABLE 2:** Encapsulation efficiency and mean diameter of the alginate (ALG) and WPI‐coated alginate (WPI/ALG) beads.

Treatments	Diameter (μm)	EE (%)
ALG bead	170^a^	88.05 ± 1.05
WPI‐coated ALG bead	250^b^	91.85 ± 0.08

*Note:* Means followed by different lowercase letters differ statistically in column *(p < 0.05)*. The values obtained are the means ± standard deviation of triplicates.

Table 2 presents the results of bacterial encapsulation efficiency in both ALG and WPI‐coated ALG beads. Both carriers demonstrated high encapsulation efficiency, with values ranging from 88.05% to 91.85%. Extrusion, an easy and inexpensive method that operates under mild conditions, reduces cell damage and enhances encapsulation efficiency (Dehkordi et al. 2020). The results suggest that the application of a WPI coating to the beads, aimed at providing additional protection to the probiotic bacteria, did not negatively impact the encapsulation yields of the probiotics. These findings are consistent with previous research conducted by Hébrard et al. (2010).

### 3.3 The Antioxidant Capacity of Sports Drink: Impact of Fermentation and Storage

**FIGURE 3:**
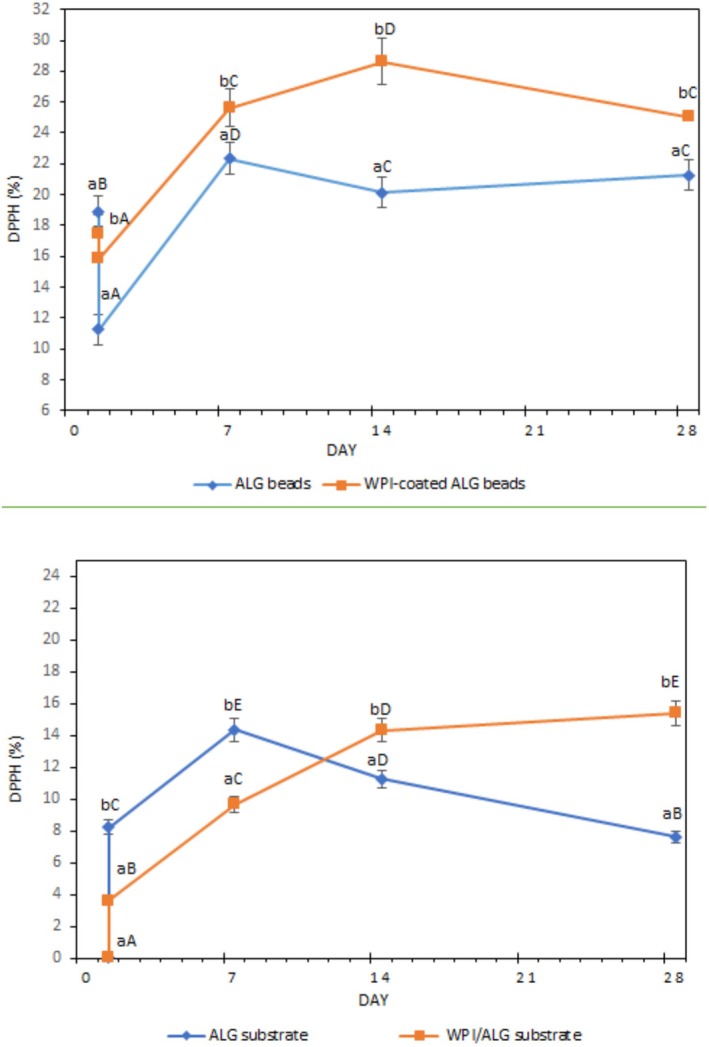
Antioxidant capacity (DPPH) of beads and substrate for sport drink samples with alginate (ALG) and WPI‐coated alginate (WPI/ALG) beads, during fermentation and 28 days of storage at 4°C. Means in a column shown with different lowercase letters are significantly different (*p* < 0.05). Means in a row shown with different uppercase letters are significantly different a row (*p* < 0.05).

Figure 3 represents the antioxidant capacity of the beads and substrates for whey permeate‐based drink samples with ALG and WPI‐coated ALG beads. The results indicate a significant decrease in antioxidant capacity for both the uncoated and WPI‐coated beads during fermentation (*p* < 0.05). This may be due to the trapped bioactive peptides and amino acids partially migrating through the porous surface of the beads to the drink samples and partially being utilized for bacterial growth inside the beads during fermentation (Kirbas and Altay 2023; Misra et al. 2021). During fermentation, the probiotic culture generates a range of bioactive molecules with antioxidant activity. However, the decrease in antioxidant capacity observed in the carriers suggests that the release of peptides into the drink samples and the consumption of peptides by probiotics surpass the production of these bioactive molecules during storage (Heydari et al. 2023).

During fermentation and storage, the ALG beads exhibited a higher decrease in antioxidant capacity compared to the WPI‐coated ALG beads (*p* < 0.05). This difference suggests that the WPI coating may have contributed to an improved controlled release of bioactive peptides from the coated beads. The WPI coating acts as a protective layer on the surface of the alginate beads, reducing their porosity and enhancing their functional barrier and protective properties (Gbassi et al. 2009).

The antioxidant capacity of the whey permeate‐based substrate was found to be significantly higher in the drink sample containing ALG beads during fermentation and the initial 7 days of storage, compared to the sample with WPI‐coated ALG beads (*p* < 0.05). During the subsequent 2 weeks of storage, a significant decline in antioxidant capacity was observed in the ALG bead sample (*p* < 0.05). The drink sample with WPI‐coated beads exhibited a delayed and controlled release of bioactive peptides from the beads to the substrate during storage. At the end of the 28‐day storage period, the sample with WPI‐coated ALG beads demonstrated a significantly higher antioxidant capacity (15.37%) compared to the sample with uncoated beads (*p* < 0.05). In a related study, it was found that the coating process of alginate hydrogel beads resulted in enhanced functionality of the samples, mainly attributed to reduced degradation of anthocyanins during storage. The protective effect of the coatings, including chitosan, WPC, or gelatin, was observed in maintaining the quality and stability of the jussara extract encapsulated within the alginate beads (Silva Carvalho et al. 2019).

### 3.4 pH and Titratable Acidity

The results presented in Table 3 demonstrate a significant decrease in pH for both drink samples during the storage period, indicating the production of lactic acid by the starter culture (*p* < 0.05). No significant difference in pH was observed between the drink samples containing uncoated and WPI‐coated beads. However, at the end of the storage period, the fermented drink sample containing WPI in the bead matrix exhibited a higher pH value compared to the sample with alginate beads. This finding is consistent with the study conducted by Peiris et al. (2019), which reported no significant difference in pH between control yogurt and yogurt containing Bifidobacterium encapsulated in an alginate‐goat milk‐inulin matrix, suggesting that the encapsulation of probiotics did not significantly affect the acidity of the fermented product. There was no significant difference in titratable acidity (*p* > 0.05) between the samples. Despite the high acidity observed in the samples with uncoated and coated ALG beads, the pH remained within the range of 4–4.5. This observation can be attributed to the buffering effect of whey peptides and amino acids (Krunić et al. 2016).

**TABLE 3:** pH and titratable acidity values of sport drink samples containing alginate (ALG) and WPI‐coated alginate (WPI/ALG) beads during refrigerated storage.

Treatment	Storage	Drink sample/ALG	Drink sample/WPI Coated ALG
pH	1st day	4.81 ± 0.02^d^	4.84 ± 0.19^d^
	7th day	4.69 ± 0.15^c^	4.72 ± 0.12^c^
	14th day	4.61 ± 0.06^bc^	4.69 ± 0.23^b^
	21st day	4.42 ± 0.06^b^	4.51 ± 0.06^b^
	28th day	4.10 ± 0.12^a^	4.21 ± 0.15^a^
	1st day	4.81 ± 0.02^d^	4.84 ± 0.19^d^
Acidity (%Acid lactic)	1st day	1.21 ± 1.09^a^	1.24 ± 1.09^a^
	7th day	1.31 ± 0.09^ab^	1.29 ± 0.08^ab^
	14th day	1.35 ± 0.03^ab^	1.32 ± 0.15^ab^
	21st day	1.66 ± 0.03^b^	1.70 ± 0.15^b^
	28th day	2.54 ± 0.10^c^	2.51 ± 0.10^c^

*Note:* Numbers are expressed as mean ± standard deviation. Means in a column shown with different lowercase letters are significantly different (*p* < 0.05).

### 3.5 Viability of Encapsulated and Released Probiotic Starter Culture Cells During Storage

**FIGURE 4:**
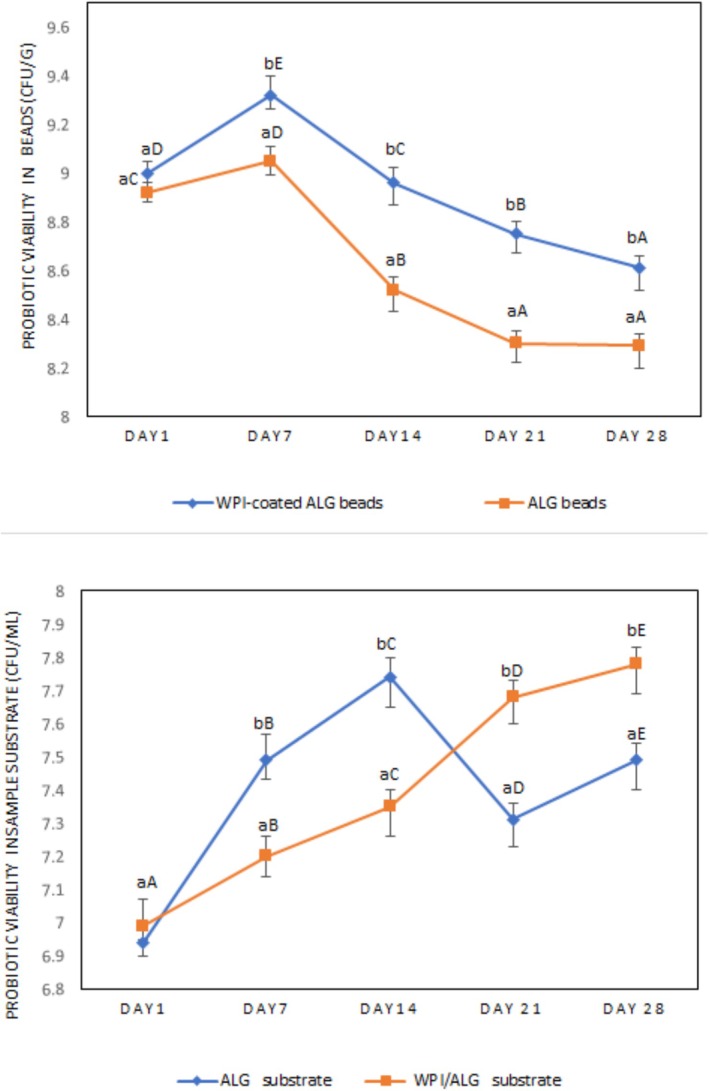
Viability of probiotic bacteria for encapsulated cells (Log cfu/g) and released cells (Log cfu/ml) in sport drink samples with alginate (ALG) and WPI‐coated alginate (WPI/ALG) beads, during 28 days of storage at 4°C. Means in a column shown with different lowercase letters are significantly different (*p* < 0.05). Means in a row shown with different uppercase letters are significantly different (*p* < 0.05).

The viability of encapsulated probiotics during storage and under hostile conditions is influenced by the type of probiotic bacteria, the methods of encapsulation, and the materials used for coating (Safeer Abbas et al. 2023). In this study, the growth of the encapsulated probiotic culture significantly increased during the first 7 days of storage in both ALG and WPI‐coated ALG beads (see Figure 4; *p* < 0.05). The notable growth observed in the beads containing WPH indicates that the bioactive peptides within the microbeads may positively influence bacterial growth. This suggests that these peptides could function as prebiotics, promoting growth and enhancing metabolism, which warrants further investigation. Previous research has also demonstrated that whey peptides can improve probiotic growth (Dave and Shah 1998; Yu et al. 2016).

At the end of the storage period (day 28), both drink samples containing coated and uncoated beads exhibited viable counts exceeding the recommended level of 6 log CFU/g (Nyanzi et al. 2021). The drink samples with WPI‐coated beads displayed significantly higher probiotic counts (8.61 ± 0.14 log CFU/g) after 28 days of storage compared to those with uncoated beads (*p* < 0.05). These findings suggest that the addition of WPI coating resulted in a more stable matrix with increased acid tolerance, likely due to the high buffering properties of WPI (Shi et al. 2013). The buffering capacity to stabilize pH is a crucial aspect for commercial exploitation as it ensures the bacteria remain intact in the food until consumption (Gbassi et al. 2009). The protective effect of whey protein in the encapsulation of probiotics has been consistently highlighted in previous research (Chen and Subirade 2006).

The cell viability of released probiotics was evaluated in substrates containing both coated and uncoated beads, as shown in Figure 4. The viability of probiotic‐free cells released from beads to the substrate was significantly higher for the drink sample with uncoated beads compared to the drink sample with WPI‐coated beads during the first 14 days (*p* < 0.05). This could be attributed to the higher porosity of alginate particles, which facilitated the release of a larger portion of their content compared to coated microparticles. The viability of released probiotic free cells decreased for the drink sample with uncoated beads in the next 2 weeks. The drink sample with WPI‐coated ALG beads exhibited a controlled and gradual increase in the number of released probiotic cells. The whey protein coating's protective effect enables a more controlled release of probiotics.

In a study conducted by Gaudreau et al. (2016), pectinate microbeads coated with whey protein were employed to safeguard probiotic cells. The results of their study corroborated the notion that coating alginate microbeads augments probiotic viability during product storage. This finding aligns with an earlier study by Doherty et al. (2012), which observed complete probiotic mortality when probiotic cells were suspended in alginate, while coated alginate microbeads were found to enhance probiotic viability.

### 3.6 Survival Rate of Free and Encapsulated Probiotics During In Vitro Digestion

**FIGURE 5:**
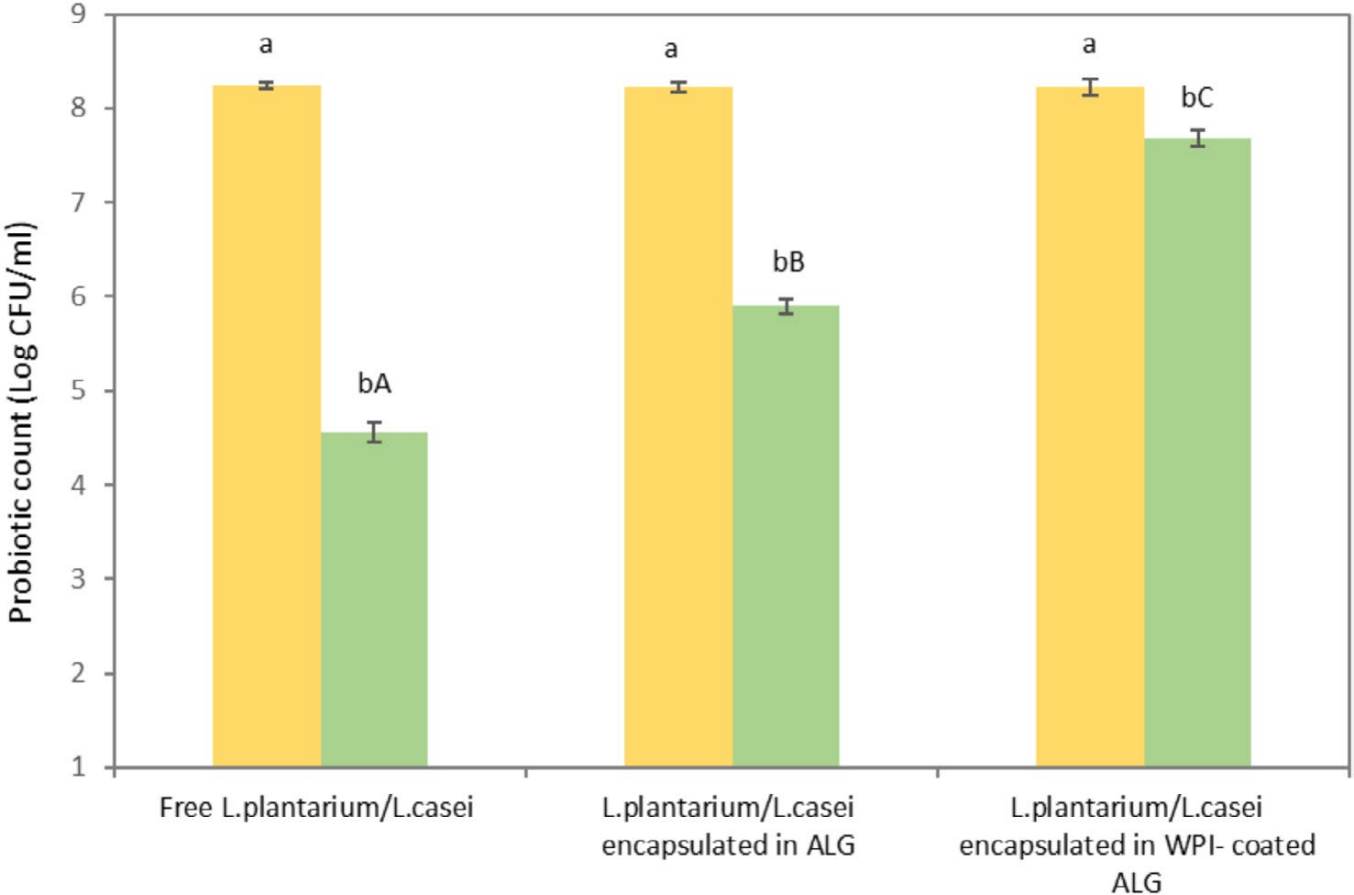
Free and encapsulated probiotic cells’ survival under the simulated gastric condition. Means followed by different lowercase letters differ statistically in column (*p* < 0.05).

The results, as depicted in Figure 5, present the viability of free and encapsulated probiotic cells following a 2‐h digestion period in simulated gastric fluid (SGF) at pH 2.0. The findings reveal a significant difference in the log CFU/g reduction among the experimental groups (*p* < 0.05). Notably, the reduction values observed were 3.68, 2.32, and 0.54 for free probiotic cells, cells encapsulated within ALG beads, and cells encapsulated within WPI‐coated ALG beads, respectively.

The study findings suggest that the co‐encapsulation of probiotics and WPH can alleviate the acid‐induced harm to *L. plantarum* and *L. casei* cells under low pH conditions. This protective effect could be attributed to the presence of bioactive peptides in whey, which act as antiradical agents. These agents can moderate the oxidative stress induced by cellular metabolic activities, thereby creating a more conducive environment for cell proliferation under acidic conditions. Furthermore, the buffering capacity of WPH in acidic media was found to counteract the adverse effects of acid on probiotics (Ilie et al. 2022).

In this regard, Huang et al. (2023), investigated the use of osteopontin (OPN) in pectin‐based microencapsulation to enhance the viability of Bifidobacteria during processing and simulated infant gastrointestinal digestion. Their study found that adding OPN significantly improved the survival of *Bifidobacterium bifidum* and *Bifidobacterium breve* in adverse conditions, such as freeze‐drying and digestion. Gaudreau et al. (2016) reported that the co‐encapsulation of bacteria with green tea extract as a natural antioxidant provided additional protection to the cells during gastric conditions.

In another study, Ejaz et al. (2023) investigated the survival and sustainability of a *Lactobacillus rhamnosus* probiotic encapsulated in microcapsules. The prebiotic impact of two different kinds of onion (*Allium cepa L*.) peel extract was also examined. The study found that the effective inclusion of red and white onion peel extract into the microcapsules enhanced the survival of the probiotic. Furthermore, the encapsulated probiotics exhibited significantly greater vitality compared to free cells, especially under simulated gastrointestinal conditions.

The encapsulation of probiotics in WPI‐coated beads showed better survivability compared to uncoated beads. This disparity in viability can be attributed to the characteristics of alginate, which exhibits poor resistance to low pH and is prone to dissolution in the presence of ions or monovalent salts that bind calcium ions (Goderska and Kozłowski 2021). The researchers hypothesized that the formation of a cohesive network upon coating with WPI may have reduced the penetration of H+ into the beads, leading to increased stability and relatively gentle cell reduction. This effect could be attributed to the extraordinary acidic tolerance and high buffering properties of WPI (Razavi et al. 2021). The enzyme‐resistant globular protein fractions of WPI, such as β‐lactoglobulin, can also protect WPI‐coated beads and attenuate the negative effect of gastric juice on the encapsulated probiotics due to their particular folded calyx structure, where the target amino acid residues are buried in the protein core (Feng et al. 2022). Similarly, Gbassi et al. (2009) reported enhanced survival of *Lactobacillus plantarum* spp. through the whey protein coating of alginate beads.

Furthermore, the difference in efficacy in probiotic cell protection between ALG beads and WPI‐coated beads can be attributed to the higher release rate of WPH under simulated gastrointestinal conditions (SGC) in uncoated ALG beads relative to WPI‐coated beads. As a result, the higher release rate of WPH results in a less supportive effect on the viability of the cells (Han et al. 2020; Vargas et al. 2015). In this regard, Silva Carvalho et al. (2019) found that the Alginate microparticles containing jussara (*Euterpe edulis* Martius) extract released 76% of anthocyanins in the simulated gastric phase, while microparticles coated with WPC released 71%. Gaudreau et al. (2016) proposed that whey protein‐coated pectinate microparticles can effectively serve as a carrier for the simultaneous delivery of natural antioxidants, such as green tea extract, and probiotics to the lower regions of the gastrointestinal tract. In another study, Guo et al. (2023) studied the survival thermoresistance of *Lactobacillus rhamnosus GG* (L.GG) and inulin co‐encapsulated in alginate/pectin composite beads. They found that the co‐encapsulation process improved the survival of *L.GG* during encapsulation, storage, and in vitro digestion, with the highest viability observed after 60‐day storage and under specific heat conditions.

### 3.7 Sensory Evaluation

In Table 4, the sensory evaluation scores of sports drink samples are presented over a 28‐day storage period. The panelists observed a statistically significant sensorial change in the treatments during this period (*p < 0.05*). The sensory scores decreased for both drink samples containing uncoated and WPI‐coated ALG beads. An increase in acidity during storage led to a reduction in sensory scores, particularly for odor, due to the acidic smell of the treatments.

**TABLE 4:** Predominant sensory scores (1–5) * for taste, odor, thirst quenching, and overall acceptability of sport drink samples containing alginate (ALG) and WPI‐coated alginate (WPI/ALG) beads during 28 days of storage at 4°C.

Parameter	Group	Storage time				
		1	7	14	21	28
Taste	Drink sample/ALG	5 ± 0.00^D^	4.10 ± 0.23^aC^	4 ± 0.00^aC^	3.5 ± 0.00^aB^	3 ± 0.00^aA^
	Drink sample/WPI‐coated ALG	5 ± 0.00^D^	5 ± 0.00^bD^	4.70 ± 0.55^bC^	4.52 ± 0.85^bB^	4.01 ± 0.08^bA^
Odor	Drinksample/ALG	5 ± 0.00^D^	4.82 ± 0.23^D^	4 ± 0.00^aC^	3.6 ± 0.83^aB^	3 ± 0.00^aA^
	Drinksample/WPI‐coated ALG	5 ± 0.00^C^	4.80 ± 0.00^C^	4.56 ± 0.21^bB^	4.22 ± 0.24^bA^	3.99 ± 0.82^bA^
Thirst quenching	Drink sample/ALG	5 ± 0.00^B^	5 ± 0.00^B^	4.80 ± 0.21^B^	4.20 ± 0.21^aA^	4.00 ± 0.21^A^
	Drink sample/WPI‐coated ALG	5 ± 0.00^B^	5 ± 0.00^B^	5.00 ± 0.21^B^	4.60 ± 0.21^bA^	4.22 ± 0.21^A^
Overall Acceptability	Drink sample/ALG	5 ± 0.00^D^	4.5 ± 0.00^aD^	4 ± 0.00^aC^	3.54 ± 0.16^aB^	3.25 ± 0.23^aA^
	Drink sample/WPI‐coated ALG	5 ± 0.00^C^	5 ± 0.00^bC^	4.77 ± 0.16^bB^	4.50 ± 0.33^bAB^	4.20 ± 0.16^bA^

*Note:* Data are means ± SD. Means in a column shown with different lowercase letters are significantly different (*p* < 0.05). Means in a row shown with different uppercase letters are significantly different (*p* < 0.05).

The panelists reported a bitter taste after 7 days for drink samples containing uncoated ALG beads. This could be attributed to the release of bioactive peptides during storage, causing a negative sensory experience for the panelists after 7 days. By contrast, WPI‐coated beads delayed the onset of bitterness until day 21, presumably through a slower release of peptides.

In this regard, Rao et al. (2016) observed a significant reduction in bitterness with higher concentrations of encapsulation material for casein peptides. Similarly, Sarabandi et al. (2018) found that microencapsulation via spray‐drying mitigated the bitter taste of casein peptides in a pastille formulation. Another study conducted by Ma et al. (2014) showed that encapsulated whey peptides via spray‐drying and freeze‐drying exhibited lower bitterness scores than free peptides. Moreover, Yang et al. (2012) revealed that whey peptides had less bitterness after encapsulation with MD or the MD/β‐cyclodextrin mixture via spray‐drying.

The panelists reported both drink samples to have a salty taste but found them acceptable in terms of thirst‐quenching. The salty taste intensity of WP drinks was not unpleasant for the panelists, indicating that electrolytes were in balance and in adequate proportion. The objective of the study was to design a sports drink from WP that aligns with the concept of thirst‐quenching beverages. To achieve this, it was important to formulate a drink with an adequate concentration of minerals to ensure proper fluid absorption and rehydration as well as maintain the perception of thirst‐quenching.

However, due to variations in minerals resulting from factors such as cheese type, season, feed type, and dairy cow genetics, regular analysis and standardization of the permeate are necessary to ensure compliance with legislation (sodium should be below the limit established by legislation, which imposes a maximum of 700 mg/L) and acceptable sensory properties.

The information provided indicates that encapsulation technology offers a viable means of addressing the difficulties posed by bioactive peptides that have impeded their use in nutraceutical and commercial applications.

## 4 Conclusion

In this study, we formulated a whey permeate‐based sports drink containing co‐encapsulated probiotics and WPH. The results showed enhanced probiotic viability, antioxidant capacity, and sensory properties of the formulated drink. Utilizing whey permeate, a by‐product of the dairy industry, not only makes this product economically viable but also contributes to sustainable production practices by minimizing waste. However, a limitation of this study was the financial constraint that precluded an evaluation of the in vivo effects of these beverages on hydroelectrolytic replenishment and sports performance. Further research is necessary to explore this aspect.

## Author Contributions

**Maryam Soltani:** data curation (equal), formal analysis (equal), investigation (equal), software (equal), writing – review and editing (equal). **Mahdieh Pabast:** conceptualization (equal), methodology (equal), validation (equal).

## Funding

This work was supported by Islamic Azad University.

## Conflicts of Interest

The authors declare no conflicts of interest.

## Data Availability

All data generated or analyzed during this study are included in this published article.

## References

[fsn371255-bib-0002] Afzaal, M. , F. Saeed , H. Ateeq , et al. 2023. “Probiotics Encapsulated Gastroprotective Cross‐Linked Microgels: Enhanced Viability Under Stressed Conditions With Dried Apple Carrier.” Food Science & Nutrition 11, no. 2: 817–827. 36789050 10.1002/fsn3.3116 PMC9922151

[fsn371255-bib-0003] Balawejder, M. , M. Szostek , J. Gorzelany , P. Antos , G. Witek , and N. Matłok . 2020. “A Study on the Potential Fertilization Effects of Microgranule Fertilizer Based on the Protein and Calcined Bones in Maize Cultivation.” Sustainability 12, no. 4: 1343.

[fsn371255-bib-0004] Berry, C. W. , B. Murray , and W. L. Kenney . 2022. “Scientific Basis for a Milk Permeate‐Based Sports Drink–A Critical Review.” International Dairy Journal 127: 105296.

[fsn371255-bib-0006] Burgain, J. , C. Gaiani , M. Linder , and J. Scher . 2011. “Encapsulation of Probiotic Living Cells: From Laboratory Scale to Industrial Applications.” Journal of Food Engineering 104, no. 4: 467–483.

[fsn371255-bib-0007] Chen, L. , and M. Subirade . 2006. “Alginate–Whey Protein Granular Microspheres as Oral Delivery Vehicles for Bioactive Compounds.” Biomaterials 27, no. 26: 4646–4654. 16714058 10.1016/j.biomaterials.2006.04.037

[fsn371255-bib-0008] Dave, R. I. , and N. P. Shah . 1998. “Ingredient Supplementation Effects on Viability of Probiotic Bacteria in Yogurt.” Journal of Dairy Science 81, no. 11: 2804–2816. 9839222 10.3168/jds.S0022-0302(98)75839-4

[fsn371255-bib-0011] Dehkordi, S. S. , I. Alemzadeh , A. S. Vaziri , and A. Vossoughi . 2020. “Optimization of Alginate‐Whey Protein Isolate Microcapsules for Survivability and Release Behavior of Probiotic Bacteria.” Applied Biochemistry and Biotechnology 190: 182–196. 31313242 10.1007/s12010-019-03071-5

[fsn371255-bib-0013] Doherty, S. B. , M. A. Auty , C. Stanton , R. P. Ross , G. F. Fitzgerald , and A. Brodkorb . 2012. “Application of Whey Protein Micro‐Bead Coatings for Enhanced Strength and Probiotic Protection During Fruit Juice Storage and Gastric Incubation.” Journal of Microencapsulation 29, no. 8: 713–728. 22970750 10.3109/02652048.2011.638994

[fsn371255-bib-0014] Drgalic, I. , L. Tratnik , and R. Bozanic . 2005. “Growth and Survival of Probiotic Bacteria in Reconstituted Whey.” Le Lait 85, no. 3: 171–179.

[fsn371255-bib-0015] Ejaz, A. , M. Afzaal , F. Saeed , et al. 2023. “Development and Characterization of Symbiotic Microcapsules to Enhance the Viability of Probiotic Under Stressed Conditions.” International Journal of Food Properties 26, no. 2: 2838–2853.

[fsn371255-bib-0016] Feng, Y. , D. Yuan , C. Cao , et al. 2022. “Changes of In Vitro Digestion Rate and Antioxidant Activity of Digestion Products of Ethanol‐Modified Whey Protein Isolates.” Food Hydrocolloids 131: 107756.

[fsn371255-bib-0017] Gad, A. S. , W. H. Emam , G. F. Mohamed , and A. F. Sayd . 2013. “Utilization Whey in Production of Functional Healthy Beverage Whey‐Mango Beverages.” American Journal of Food Technology 8, no. 3: 133–148.

[fsn371255-bib-0018] Gamage, S. M. , M. K. S. Mihirani , O. D. A. N. Perera , and H. L. Weerahewa . 2016. “Development of Synbiotic Beverage From Beetroot Juice Using Beneficial Probiotic *Lactobacillus Casei* 431.” Ruhuna Journal of Science 7, no. 2: 64–69.

[fsn371255-bib-0019] Gao, D. , Y. Cao , and H. Li . 2010. “Antioxidant Activity of Peptide Fractions Derived From Cottonseed Protein Hydrolysate.” Journal of the Science of Food and Agriculture 90, no. 11: 1855–1860. 20602516 10.1002/jsfa.4024

[fsn371255-bib-0020] Gaudreau, H. , C. P. Champagne , G. E. Remondetto , A. Gomaa , and M. Subirade . 2016. “Co‐Encapsulation of *Lactobacillus helveticus* Cells and Green Tea Extract: Influence on Cell Survival in Simulated Gastrointestinal Conditions.” Journal of Functional Foods 26: 451–459.

[fsn371255-bib-0021] Gbassi, G. K. , T. Vandamme , S. Ennahar , and E. Marchioni . 2009. “Microencapsulation of *Lactobacillus plantarum* spp in an Alginate Matrix Coated With Whey Proteins.” International Journal of Food Microbiology 129, no. 1: 103–105. 19059666 10.1016/j.ijfoodmicro.2008.11.012

[fsn371255-bib-0022] Goderska, K. , and P. Kozłowski . 2021. “Evaluation of Microencapsulated Synbiotic Preparations Containing Lactobionic Acid.” Applied Biochemistry and Biotechnology 193: 3483–3495. 34282567 10.1007/s12010-021-03622-9 PMC8536647

[fsn371255-bib-0023] Guo, Q. , B. Cui , C. Yuan , et al. 2023. “Fabrication of Dry S/O/W Microcapsule and Its Probiotic Protection Against Different Stresses.” Journal of the Science of Food and Agriculture 104, no. 5: 2842–2850. 38012057 10.1002/jsfa.13175

[fsn371255-bib-0025] Han, C. , Y. Xiao , E. Liu , Z. Su , X. Meng , and B. Liu . 2020. “Preparation of ca‐Alginate‐Whey Protein Isolate Microcapsules for Protection and Delivery of L. Bulgaricus and *L. paracasei* .” International Journal of Biological Macromolecules 163: 1361–1368. 32745547 10.1016/j.ijbiomac.2020.07.247

[fsn371255-bib-0026] Hébrard, G. , V. Hoffart , E. Beyssac , J. M. Cardot , M. Alric , and M. Subirade . 2010. “Coated Whey Protein/Alginate Microparticles as Oral Controlled Delivery Systems for Probiotic Yeast.” Journal of Microencapsulation 27, no. 4: 292–302. 20163284 10.3109/02652040903134529

[fsn371255-bib-0027] Heydari, S. , S. E. Hosseini , A. M. Mortazavian , and S. Taheri . 2023. “Extraction of Bioactive Peptides Produced in Probiotic Yoghurt and Determination of Their Biological Activities.” International Dairy Journal 139: 105544.

[fsn371255-bib-0028] Huang, Y. , Z. Lu , F. Liu , et al. 2023. “Osteopontin Enhances the Probiotic Viability of Bifidobacteria in Pectin‐Based Microencapsulation Subjected to In Vitro Infant Gastrointestinal Digestion.” Food Hydrocolloids 149: 109634. 10.1016/j.foodhyd.2023.109634.

[fsn371255-bib-0029] Ilie, D. , A. Iosageanu , O. Craciunescu , A. M. Seciu‐Grama , C. Sanda , and F. Oancea . 2022. “Free Radical Scavenging, Redox Balance and Wound Healing Activity of Bioactive Peptides Derived From Proteinase K‐Assisted Hydrolysis of *Hypophthalmichthys molitrix* Skin Collagen.” Food Technology and Biotechnology 60, no. 3: 281–292. 36320350 10.17113/ftb.60.03.22.7107 PMC9590264

[fsn371255-bib-0030] Karim, A. , and M. Aider . 2022. “Production of Prebiotic Lactulose Through Isomerisation of Lactose as a Part of Integrated Approach Through Whey and Whey Permeate Complete Valorisation: A Review.” International Dairy Journal 126: 105249.

[fsn371255-bib-0031] Kewuyemi, Y. O. , H. Kesa , O. A. Adebo , and A. Ayodeji . 2022. “Trends in Functional Food Development With Three‐Dimensional (3D) Food Printing Technology: Prospects for Value‐Added Traditionally Processed Food Products.” Critical Reviews in Food Science and Nutrition 62, no. 28: 7866–7904. 33970701 10.1080/10408398.2021.1920569

[fsn371255-bib-0032] Kheroufi, A. , M. E. Brassesco , D. A. Campos , H. Boughellout , and M. E. Pintado . 2022. “Functional Properties of Peptides Obtained From Whey Proteins by Ficin Extract Hydrolysis.” Food Bioscience 47: 101707.

[fsn371255-bib-0033] Kirbas, Z. , and F. Altay . 2023. “Incorporating Antioxidative Peptides Within Nanofibrous Delivery Vehicles: Characterization and in Vitro Release Kinetics.” Food Bioscience 53: 102660.

[fsn371255-bib-0035] Krunic, T. , M. Rakin , M. Bulatovic , and D. Zaric . 2018. “The Contribution of Bioactive Peptides of Whey to Quality of Food Products.” In Food Processing for Increased Quality and Consumption, 251–285. Elsevier.

[fsn371255-bib-0036] Krunić, T. Ž. , M. L. Bulatović , N. S. Obradović , M. S. Vukašinović‐Sekulić , and M. B. Rakin . 2016. “Effect of Immobilisation Materials on Viability and Fermentation Activity of Dairy Starter Culture in Whey‐Based Substrate.” Journal of the Science of Food and Agriculture 96, no. 5: 1723–1729. 26033314 10.1002/jsfa.7278

[fsn371255-bib-0037] Krunić, T. Ž. , and M. B. Rakin . 2022. “Enriching Alginate Matrix Used for Probiotic Encapsulation With Whey Protein Concentrate or Its Trypsin‐Derived Hydrolysate: Impact on Antioxidant Capacity and Stability of Fermented Whey‐Based Beverages.” Food Chemistry 370: 130931. 34509939 10.1016/j.foodchem.2021.130931

[fsn371255-bib-0038] Legarová, V. , and L. Kouřimská . 2010. “Sensory quality evaluation of whey‐based beverages.” Mljekarstvo 60, no. 4: 280–287.

[fsn371255-bib-0039] Ma, J. J. , X. Y. Mao , Q. Wang , et al. 2014. “Effect of Spray Drying and Freeze Drying on the Immunomodulatory Activity, Bitter Taste and Hygroscopicity of Hydrolysate Derived From Whey Protein Concentrate.” LWT‐ Food Science and Technology 56, no. 2: 296–302.

[fsn371255-bib-0040] Mann, B. , A. Kumari , R. Kumar , et al. 2015. “Antioxidant Activity of Whey Protein Hydrolysates in Milk Beverage System.” Journal of Food Science and Technology 52: 3235–3241. 26028704 10.1007/s13197-014-1361-3 PMC4444869

[fsn371255-bib-0041] Misra, S. , P. Pandey , and H. N. Mishra . 2021. “Novel Approaches for Co‐Encapsulation of Probiotic Bacteria With Bioactive Compounds, Their Health Benefits and Functional Food Product Development: A Review.” Trends in Food Science & Technology 109: 340–351.

[fsn371255-bib-0042] Nyanzi, R. , P. J. Jooste , and E. M. J. J. Buys . 2021. “Invited Review: Probiotic Yogurt Quality Criteria, Regulatory Framework, Clinical Evidence, and Analytical Aspects.” Journal of Dairy Science 104, no. 1: 1–19. 33348476 10.3168/jds.2020-19116

[fsn371255-bib-0043] Olbrycht, M. , M. Kołodziej , R. Bochenek , et al. 2020. “Mechanism of nutrition activity of a microgranule fertilizer fortified with proteins.” Biomolecules 10, no. 1: 1–12. 10.1186/s12870-020-02340-4 PMC7092569 32209052

[fsn371255-bib-0044] Osman, A. , A.‐R. M. Merwad , A. H. Mohamed , and M. Sitohy . 2021. “Foliar Spray With Pepsin‐and Papain‐Whey Protein Hydrolysates Promotes the Productivity of Pea Plants Cultivated in Clay Loam Soil.” Journal of Materials and Environmental Science 26, no. 9: 2805. 10.3390/molecules26092805 PMC8126062 34068570

[fsn371255-bib-0045] Pedrali, D. , A. Scarafoni , A. Giorgi , and V. Lavelli . 2023. “Binary Alginate‐Whey Protein Hydrogels for Antioxidant Encapsulation.” Antioxidants 12, no. 6: 1192. 37371922 10.3390/antiox12061192 PMC10295361

[fsn371255-bib-0046] Peiris, K. R. M. , P. H. P. Prasanna , D. V. P. Chandramali , D. C. S. Gunasekara , and P. M. V. Madhusanka . 2019. Effect of Incorporation of Kithul Flour on Physical, Microbiological and Sensory Attributes of Probiotic Set Yogurt.

[fsn371255-bib-0047] Pitigraisorn, P. , K. Srichaisupakit , N. Wongpadungkiat , and S. Wongsasulak . 2017. “Encapsulation of *Lactobacillus acidophilus* in Moist‐Heat‐Resistant Multilayered Microcapsules.” Journal of Food Engineering 192: 11–18.

[fsn371255-bib-0048] Rao, P. S. , R. K. Bajaj , B. Mann , S. Arora , and S. Tomar . 2016. “Encapsulation of Antioxidant Peptide Enriched Casein Hydrolysate Using Maltodextrin‐Gum Arabic Blend.” Journal of Food Science and Technology 53: 3834–3843. 28017999 10.1007/s13197-016-2376-8 PMC5147710

[fsn371255-bib-0049] Razavi, S. , S. Janfaza , N. Tasnim , D. L. Gibson , and M. Hoorfar . 2021. “Microencapsulating Polymers for Probiotics Delivery Systems: Preparation, Characterization, and Applications.” Food Hydrocolloids 120: 106882.

[fsn371255-bib-0050] Safeer Abbas, M. , M. Afzaal , F. Saeed , et al. 2023. “Probiotic Viability as Affected by Encapsulation Materials: Recent Updates and Perspectives.” International Journal of Food Properties 26, no. 1: 1324–1350.

[fsn371255-bib-0051] Sarabandi, K. , A. S. Mahoonak , H. Hamishekar , M. Ghorbani , and S. M. Jafari . 2018. “Microencapsulation of Casein Hydrolysates: Physicochemical, Antioxidant and Microstructure Properties.” Journal of Food Engineering 237: 86–95.

[fsn371255-bib-0052] Servili, M. , C. Rizzello , A. Taticchi , et al. 2011. “Functional Milk Beverage Fortified With Phenolic Compounds Extracted From Olive Vegetation Water, and Fermented With Functional Lactic Acid Bacteria.” International Journal of Food Microbiology 147, no. 1: 45–52. 21458095 10.1016/j.ijfoodmicro.2011.03.006

[fsn371255-bib-0053] Shi, Q. , Z. Fang , and B. Bhandari . 2013. “Effect of Addition of Whey Protein Isolate on Spray‐Drying Behavior of Honey With Maltodextrin as a Carrier Material.” Drying Technology 31, no. 13–14: 1681–1692.

[fsn371255-bib-0054] Silva Carvalho, A. D. , M. D. Costa Machado , H. D. D. F. Q. Barros , C. B. B. Cazarin , and Maróstica Junior, MR, & Hubinger, MD . 2019. “Anthocyanins From Jussara (Euterpe Edulis Martius) Extract Carried by Calcium Alginate Beads Pre‐Prepared Using Ionic Gelation.” Powder Technology 345: 283–291.

[fsn371255-bib-0055] Song, L. , Y. Chen , Z. Liu , et al. 2023. “Preparation, Characterization, and Stability Assessment of a Nano‐Delivery System Loaded With Phosvitin Phosphopeptide‐Calcium Chelate.” Food Bioscience 56: 103306.

[fsn371255-bib-0056] Stokes, C. N. , M. G. O'Sullivan , and J. P. Kerry . 2017. “Hedonic and Descriptive Sensory Evaluation of Instant and Fresh Coffee Products.” European Food Research and Technology 243: 331–340.

[fsn371255-bib-0057] Varga, L. 2006. “Effect of Acacia (* Robinia pseudo‐acacia L*.) Honey on the Characteristic Microflora of Yogurt During Refrigerated Storage.” International Journal of Food Microbiology 108, no. 2: 272–275. 16478638 10.1016/j.ijfoodmicro.2005.11.014

[fsn371255-bib-0058] Vargas, L. A. , D. W. Olson , and K. J. Aryana . 2015. “Whey Protein Isolate Improves Acid and Bile Tolerances of *Streptococcus thermophilus* ST‐M5 and *Lactobacillus delbrueckii* Ssp. Bulgaricus LB‐12.” Journal of Dairy Science 98, no. 4: 2215–2221. 25622877 10.3168/jds.2014-8869

[fsn371255-bib-0059] Xia, T. , B. Zhang , W. Duan , J. Zhang , and M. Wang . 2020. “Nutrients and Bioactive Components From Vinegar: A Fermented and Functional Food.” Journal of Functional Foods 64: 103681.

[fsn371255-bib-0060] Yang, S. , X. Y. Mao , F. F. Li , et al. 2012. “The Improving Effect of Spray‐Drying Encapsulation Process on the Bitter Taste and Stability of Whey Protein Hydrolysate.” European Food Research and Technology 235: 91–97.

[fsn371255-bib-0061] Yu, Y. J. , M. Amorim , C. Marques , C. Calhau , and M. Pintado . 2016. “Effects of Whey Peptide Extract on the Growth of Probiotics and Gut Microbiota.” Journal of Functional Foods 21: 507–516.

